# Residual Traditional Risk in Non-Traditional Atherosclerotic Diseases

**DOI:** 10.3390/ijms26020535

**Published:** 2025-01-10

**Authors:** Federico Biscetti, Giorgia Polito, Maria Margherita Rando, Maria Anna Nicolazzi, Luis H. Eraso, Paul J. DiMuzio, Massimo Massetti, Antonio Gasbarrini, Andrea Flex

**Affiliations:** 1 Cardiovascular Internal Medicine Unit, Fondazione Policlinico Universitario A. Gemelli IRCCS, 00168 Roma, Italy; 2 Dipartimento di Medicina e Chirurgia Traslazionale, Università Cattolica del Sacro Cuore, 00168 Roma, Italy; 3 Division of Vascular and Endovascular Surgery, Thomas Jefferson University, Philadelphia, PA 19107, USA; 4 Dipartimento di Scienze Cardiovascolari e Pneumologiche, Università Cattolica del Sacro Cuore, 00168 Roma, Italy; 5 Department of Cardiovascular Sciences, Fondazione Policlinico Universitario A. Gemelli IRCCS, 00168 Roma, Italy; 6 Department of Internal Medicine, Università Cattolica del Sacro Cuore, 00168 Roma, Italy

**Keywords:** cardiovascular risk, atherosclerosis, residual risk, inflammation

## Abstract

Individuals with chronic inflammatory and immune disorders are at an increased risk of atherosclerotic events and premature cardiovascular (CV) disease. Despite extensive literature exploring the relationship between “non-traditional” atherosclerotic conditions and CV risk, many aspects remain unresolved, including the underlying mechanisms promoting the “non-traditional CV risk”, the development of an innovative and comprehensive CV risk assessment tool, and recommendations for tailored interventions. This review aims to evaluate the available evidence on key “non-traditional” CV risk-enhancer conditions, with a focus on assessing and managing CV risk factors. We conducted a comprehensive review of 412 original articles, narrative and systematic reviews, and meta-analyses addressing the CV risk associated with “non-traditional” atherosclerotic conditions. The analysis examined the underlying mechanisms of these relationships and identified strategies for assessing and mitigating elevated risk. A major challenge highlighted is the difficulty in quantifying the contribution of individual risk factors and disease-specific elements to CV risk. While evidence supports the cardiovascular benefits of statins beyond lipid lowering, such as pleiotropic and endothelial effects, current guidelines lack specific recommendations for the use of statins or other therapies targeting non-traditional CV risk factors. Additionally, the absence of validated cardiovascular risk scores that incorporate non-traditional risk factors hinders accurate CV risk evaluation and management. The growing prevalence of “non-traditional CV risk-enhancer conditions” underscores the need for improved awareness of CV risk assessment and management. A thorough understanding of all contributing factors, including disease-specific elements, is crucial for accurate prediction of cardiovascular disease (CVD) risk. This represents an essential foundation for informed decision-making in primary and secondary prevention. We advocate for future research to focus on developing innovative, disease-specific CV risk assessment tools that incorporate non-traditional risk factors, recognizing this as a promising avenue for translational and clinical outcome research.

## 1. Introduction

The term atherosclerotic cardiovascular diseases (ASCVDs) refers to a spectrum of conditions affecting blood vessels and the heart, characterized by the accumulation of plaque predominantly driven by lipid-related processes [1]. ASCVDs are the leading cause of mortality worldwide and a major contributor to global morbidity, accounting for nearly one-third of all annual deaths and impacting over 500 million individuals globally [2,3,4]. Over recent decades, the incidence and prevalence of ASCVDs have risen steadily, making them one of the most common chronic medical conditions worldwide [4,5]. Similar to other chronic disorders, ASCVDs not only pose significant health challenges but also impose a substantial economic burden [6]. These trends highlight the severe threat posed by cardiovascular diseases, which continue to be a critical concern for global health systems, as well as social and economic stability.

The European Society of Cardiology (ESC) guidelines identify serum apolipoprotein-B-containing lipoproteins, hypertension, diabetes mellitus, obesity, and smoking as the primary risk factors for ASCVDs [7]. Recognizing the heterogeneity of patients, current guidelines emphasize the use of specific biomarkers (e.g., low-density lipoprotein, glycated hemoglobin, body mass index) to aid in individualized risk assessment, guide management strategies, and intensify treatment when necessary [7]. However, translating cardiovascular disease risk into treatment thresholds remains challenging. No fixed threshold mandates treatment for cardiovascular (CV) risk, nor does any lower limit exclude intervention for risk factors [7]. Given the pandemic scale of cardiovascular disease (CVD), a holistic approach that extends beyond lifestyle modifications and therapeutic targets is essential. This underscores the importance of early initiation and titration of pharmacological prevention strategies, such as aspirin and lipid-lowering therapies [8,9,10,11].

The formation and progression of atherosclerotic plaques are primarily driven by the deposition of low-density lipoprotein cholesterol (LDL-C) and other apolipoprotein-B-containing lipoproteins within the arterial walls [7,12]. Consequently, lipid-lowering therapy is fundamental for reducing serum cholesterol levels, stabilizing atherosclerotic plaques, and preventing their progression or rupture. Currently, lipid-lowering therapies are the cornerstone of both primary and secondary prevention of ASCVDs [13,14]. Guidelines utilize LDL-C as a key biomarker for risk stratification and treatment adjustments, with progressively lower therapeutic targets recommended for individuals at higher risk [7,15]. Emerging evidence suggests that reducing LDL-C levels below the current targets provides additional benefits, paving the way for further refinement of lipid-lowering strategies [16].

Lifestyle interventions remain pivotal; however, pharmacological management of dyslipidemia has long relied on inhibitors of 3-hydroxy-3-methylglutaryl-coenzyme A (HMG-CoA) reductase, commonly known as statins [17]. Statins not only effectively lower LDL-C levels but also exhibit pleiotropic effects, including anti-inflammatory and antioxidant properties, offering added cardiovascular benefits [18,19]. Nonetheless, many patients struggle to achieve optimal LDL-C targets or tolerate the high statin doses often required [17].

To address these limitations, the spectrum of available lipid-lowering therapies has expanded beyond statins. Options now include cholesterol absorption inhibitors (e.g., ezetimibe), cholesterol synthesis inhibitors (e.g., bempedoic acid), bile acid sequestrants, proprotein convertase subtilisin kexin 9 (PCSK9) inhibitors (e.g., evolocumab, alirocumab), interfering RNA molecules (e.g., inclisiran), and triglyceride-lowering agents (e.g., icosapent ethyl) [15,20]. These therapies aim to meet the increasingly stringent LDL-C targets while accommodating patients who are statin intolerant or resistant to standard regimens [20].

However, these advances represent only part of the challenge. The ESC guidelines also highlight a range of “non-traditional” atherosclerotic conditions associated with elevated CV risk [15,21]. These include autoimmune, auto-inflammatory, neoplastic, and infectious diseases, each presenting unique therapeutic challenges. In some cases, the underlying disease accelerates the atherosclerotic process, while in others, standard treatments may inadvertently exacerbate ASCVD risk [15,21]. Current cardiovascular risk scores often underestimate risk in these populations, leading to suboptimal stratification and management strategies [22,23,24,25,26,27,28,29]. Therefore, with the growing prevalence and spread of these non-traditional atherosclerotic conditions, even among individuals traditionally considered part of the “ASCVD population”, addressing these gaps is critical.

This review focuses on key immune-mediated conditions and HIV infection, examining their inflammatory risk profiles and the necessity for long-term therapies in affected individuals. By highlighting these challenges, we aim to emphasize the need for more tailored prevention and management strategies, paving the way for improved outcomes in this diverse patient population.

## 2. Rheumatoid Arthritis

Rheumatoid arthritis (RA) is a systemic autoimmune and inflammatory disorder characterized by the progressive degeneration of synovial membranes in joints, leading to joint damage and deformities [30]. The emerging concept of “vascular rheumatology” highlights the critical role of chronic inflammatory burden (CIB) in promoting atherosclerotic processes in RA and similar conditions [31,32,33]. This inflammatory state is associated with pro-atherosclerotic alterations in lipoprotein profiles (e.g., elevated triglycerides and lipoprotein (a), reduced high-density lipoprotein-cholesterol), disruptions in cholesterol trafficking, increased oxidative stress, and endothelial dysfunction. As a result, RA patients face significantly heightened cardiovascular (CV) risk, contributing to increased morbidity and mortality [34,35,36].

Despite recognizing RA patients as a high-risk group for ASCVDs, current guidelines often underestimate their CV risk and fail to appropriately stratify patients into suitable risk categories [22,34]. Tools like the Cardiovascular Risk Score (QRISK2), the Expanded CVR Score for RA, the TransAtlantic Cardiovascular Risk Calculator for Rheumatoid Arthritis (ATACC-RA), and the Reynolds Score, as well as traditional risk calculators adjusted with a risk coefficient, have shown inconsistencies in estimating CV risk in this population [22,23].

The ESC position paper on lipid management in RA emphasizes the importance of stratifying patients based on disease-specific factors such as activity and severity, categorizing them into low-risk RA (LR-RA) and high-risk RA (HR-RA) groups [22]. For HR-RA patients, reclassification into higher ESC CV risk categories is recommended, with corresponding adjustments to low-density lipoprotein-cholesterol (LDL-C) targets to reflect their elevated risk [22].

The European League Against Rheumatism (EULAR) guidelines advocate for statins as the first-line lipid-lowering therapy in RA when optimal lipid control cannot be achieved through non-pharmacological measures [33,34]. Randomized controlled trials have demonstrated that statins not only improve lipid profiles but also reduce the incidence of major cardiovascular events in RA patients [37,38]. Beyond LDL-C reduction, statins exhibit anti-inflammatory, antithrombotic, and antioxidant effects, acting directly on atherosclerotic plaques and vessel walls, which contribute to their cardioprotective properties [39,40].

Thanks to their pleiotropic effects, statins are increasingly viewed not only as CV risk modifiers but also as adjunctive therapies for RA disease control [38,41,42,43]. For patients who fail to reach LDL-C targets or cannot tolerate statins, non-statin therapies such as ezetimibe and PCSK9 inhibitors offer effective alternatives [34]. Ezetimibe, in addition to inhibiting cholesterol absorption, has demonstrated anti-inflammatory properties and potential benefits in reducing RA disease activity, akin to statins [44]. Low levels of PCSK9, correlated with higher remission rates in RA patients receiving anti-tumor necrosis factor (TNF)-α therapy, further suggest a role for PCSK9 inhibitors in managing disease activity and CV risk [45].

The interplay between inflammation and CV risk also shapes the effects of antirheumatic drugs on lipid profiles and CV outcomes [34]. Traditional disease-modifying antirheumatic drugs (DMARDs), including methotrexate (MTX), hydroxychloroquine (HCQ), and sulfasalazine (SSZ), possess anti-inflammatory properties and have demonstrated protective effects against cardiovascular disease (CVD) [34,46,47]. Among biologics, anti-TNF-α agents and interleukin-6 (IL-6) inhibitors have been associated with changes in lipid levels, while the Janus kinase (JAK) inhibitor tofacitinib has shown similar effects [46,48,49,50,51]. Importantly, none of these treatments have been linked to increased CV risk; on the contrary, evidence suggests they reduce CV morbidity and events [52,53,54,55]. Limited data exist for other biologics, such as rituximab, abatacept, and anakinra, though rituximab has shown some benefits on lipid profiles [56,57].

Despite the growing understanding of RA-associated CV risk, optimal management remains challenging. Accurately quantifying disease-related risk and appropriately stratifying patients are ongoing issues. Encouragingly, the protective effects of DMARDs on CV risk suggest that suppressing inflammation outweighs the potential drawbacks of lipid changes. Furthermore, the pleiotropic benefits of lipid-lowering therapies like statins highlight their critical role in reducing CV risk while contributing to comprehensive disease control (Table 1).

## 3. Systemic Lupus Erythematosus

Systemic lupus erythematosus (SLE) is a chronic autoimmune disease characterized by inflammation of connective tissues, capable of affecting nearly any organ or system in the body [58]. Cardiovascular involvement is a well-recognized complication of SLE [59], driven by both traditional and disease-specific risk factors, including prolonged disease duration, active disease, organ damage, autoantibodies, and medications [60,61]. Dyslipidemia in SLE, marked by low and dysfunctional high-density lipoproteins (HDL), elevated triglycerides, and oxidized LDL-C, accelerates atherosclerosis and intensifies autoimmune and inflammatory responses, negatively influencing long-term outcomes [61,62,63,64]. Accelerated atherosclerosis increases the prevalence of peripheral artery disease (PAD) and premature coronary artery calcification, significantly contributing to morbidity and mortality [65,66,67,68,69].

SLE is considered an independent risk factor for CVD and is treated as a “CVD risk equivalent” due to the elevated cardiovascular morbidity and mortality observed in patients, beyond what traditional risk factors alone can explain [70]. This underscores the urgent need to enhance risk estimation models by incorporating biomarkers and disease-specific factors [23]. Early approaches, such as doubling the Framingham Risk Score (FRS), failed to capture disease activity-related heterogeneity, limiting their sensitivity for high-risk individuals [23,71,72,73]. A new SLE-specific risk score, incorporating variables like global activity score, lupus anticoagulant, and low complement C3, has been proposed to address these gaps [73]. Despite limitations in evidence, EULAR guidelines emphasize identifying and managing traditional risk factors in SLE patients [71].

While an SLE diagnosis alone does not mandate treatment, EULAR guidelines recommend lipid-lowering therapy for primary prevention according to general population guidelines [71]. Emerging evidence supports the use of statins in SLE patients, demonstrating favorable effects on CV risk, morbidity, and mortality [23,55,74,75,76,77,78]. Beyond modulating lipid profiles, statins mitigate SLE-associated atherogenesis by improving endothelial function, reducing carotid intima-media thickness, and exerting immunomodulatory effects [75,79,80,81,82,83]. However, the impact of statins on SLE disease activity remains unclear, with studies yielding inconsistent findings [76]. Observed reductions in high-sensitivity C-reactive protein (hs-CRP), tumor necrosis factor receptor levels, and antiphospholipid antibodies suggest potential benefits for disease activity [74,76,84,85].

The interplay between inflammation and atherosclerosis in SLE highlights the potential of targeting specific inflammatory pathways to reduce cardiovascular risk in SLE patients [86,87,88]. Rituximab, a B-cell-targeted therapy, has shown promise in improving lipid profiles by reducing inflammation [89,90]. Additionally, targeting the interferon-1 (INF-1) pathway, a key contributor to atherosclerosis, with novel therapies may help control disease activity while improving cardiovascular outcomes [91,92,93,94].

Given SLE’s classification as a CV risk equivalent, preventive measures and aggressive therapeutic interventions must be integral to disease management. As research evolves, focusing on immune and inflammatory mediators as potential therapeutic targets offers promising avenues for preventing and treating cardiovascular complications in SLE patients (Table 2).

## 4. Systemic Sclerosis

Systemic sclerosis (SSc) is a chronic autoimmune disorder characterized by excessive collagen deposition, leading to widespread tissue fibrosis and vasculopathy [96,97]. While microvascular dysfunction is a hallmark of SSc, the extent and impact of macrovascular disease, commonly observed in other autoimmune rheumatic conditions, are less well defined [98,99]. Nonetheless, the increasing prevalence of cardiovascular-related deaths and premature mortality among SSc patients in recent decades has raised concerns about an elevated risk of CVD in this population [98,99,100,101].

Epidemiological studies have documented a higher prevalence of macrovascular conditions, including PAD, cerebrovascular disease, and coronary artery disease, in SSc patients [102,103,104,105,106,107,108,109,110,111]. However, unlike other autoimmune diseases where accelerated atherosclerosis predominantly drives cardiovascular risk, SSc involves a complex interplay of microvascular and macrovascular pathologies. This dual involvement complicates the understanding of the mechanisms underlying CVD in SSc [99,106].

Given this complexity, a comprehensive approach to cardiovascular risk assessment is essential. This includes consideration of “non-traditional” factors such as microvascular dysfunction, which may significantly contribute to CVD development and progression in SSc patients [24]. Despite this, current guidelines recommend using general population-based tools for cardiovascular risk stratification, with the management of traditional risk factors, such as dyslipidemia, following standard guidelines for the general population [71].

Endothelial injury plays a central role in both atherosclerosis and the vasculopathy associated with SSc, driving fibrosis and pathological collagen deposition [112,113]. Evidence suggests that statins may offer benefits in mitigating endothelial dysfunction, although their specific therapeutic role in SSc remains unclear [114,115,116,117].

The unique pathophysiological characteristics of SSc present challenges in accurately estimating cardiovascular risk and stratifying patients appropriately. This gap highlights the need for tailored recommendations for cardiovascular prevention and risk factor management in SSc. Further research is warranted to explore the potential of statins as a disease-modifying therapy, particularly their ability to address both endothelial dysfunction and the fibrotic processes inherent to SSc (Table 3).

## 5. Psoriasis and Psoriatic Arthritis

Psoriasis, a chronic immune-mediated inflammatory disorder primarily affecting the skin and its appendages, has evolved from being considered solely a dermatological condition [118] to being recognized as a multisystem disease with a wide range of extracutaneous manifestations. These include psoriatic arthritis (PsA), inflammatory bowel disease (IBD), other immune disorders, and CVD [119,120,121].

Patients with psoriasis and PsA exhibit a significantly higher prevalence of CVD compared to the general population [122,123,124,125]. This increased risk is partially explained by traditional cardiovascular risk factors, such as diabetes, dyslipidemia, obesity, hypertension, metabolic syndrome, and smoking [126,127,128,129,130,131,132,133,134,135,136,137,138,139]. Moreover, psoriasis itself has emerged as an independent risk factor for CVD, with a severity-dependent relationship demonstrated in several studies [118,138,140,141,142]. Patients with severe psoriasis, as assessed by measures like the psoriasis area severity index (PASI) or body surface area (BSA), show a higher prevalence of hypertension, dyslipidemia, diabetes, metabolic syndrome, and major adverse cardiovascular events (MACE) [136,143,144,145,146].

The relationship between psoriasis and CVD is deeply intertwined, with shared immune-mediated inflammatory mechanisms playing a key role. Emerging evidence points to the involvement of IL-17 cytokines as a critical link [147,148], alongside other mechanisms such as cytokine dysregulation, platelet hyper-responsiveness, oxidative stress, endothelial dysfunction, and disruptions in skin barrier integrity [148,149,150].

The Joint American Academy of Dermatology (AAD) recommends comprehensive cardiovascular risk assessment for all psoriasis patients, advocating for the inclusion of both traditional and non-traditional risk factors. The AAD highlights the need for more frequent screening and adjustments to risk-scoring tools, particularly for patients undergoing systemic treatments or with a BSA > 10% [26,121]. In contrast, the American Heart Association (AHA) suggests evaluating subclinical atherosclerosis and biomarkers for enhanced risk assessment without establishing specific psoriasis severity thresholds [151,152]. According to the AHA guidelines, incorporating psoriasis as a risk factor may influence preventive strategies, including the initiation or intensification of statin therapy [151,153,154].

ESC and EULAR take a slightly different approach, recommending a multiplication factor of 1.5 to general population-based risk scores for patients with psoriasis, thereby promoting more standardized and consistent management [153,154].

Statins, with their pleiotropic and anti-inflammatory properties, hold promise for influencing psoriasis activity and severity [26]. By modulating vascular endothelial growth factor (VEGF), statins may inhibit vascular proliferation, a hallmark of psoriasis [155]. Some studies report improvements in psoriasis severity and progression with statin use [156,157,158,159], but others have observed no benefit or even worsening of psoriatic lesions [160,161,162,163,164,165,166]. This inconsistency underscores the need for further research into the role of lipid-lowering therapy in managing psoriasis and PsA.

Conversely, anti-psoriatic drugs are being actively investigated for their potential cardiovascular benefits. Non-biologic therapies like methotrexate [167,168] and apremilast [169,170] have demonstrated reductions in cardiovascular risk and CVD incidence. Among biologic therapies, TNF-α inhibitors have shown various cardioprotective effects, including improvements in cardiovascular biomarkers [171,172,173], traditional CV risk factors [169], and a reduced risk of MACE [174,175,176,177,178]. JAK inhibitors have shown minimal effects on MACE and no adverse impact on CV risk factors [179]. However, IL-12 and IL-23 inhibitors have yielded inconsistent results, raising concerns about their safety profiles [169,180].

Although IL-17 inhibitors were initially anticipated to have significant cardiovascular benefits due to the central role of the Th17 pathway in both psoriatic pathogenesis and CVD, recent evidence suggests that these agents do not substantially reduce the risk of cardiovascular events [181]. However, Elnabawi et al. [182] suggest that anti-IL-17 agents may offer the most favorable effects on plaque burden among biologic therapies, highlighting their potential for broader applications. Despite the growing recognition of the association between psoriasis and CVD, significant knowledge gaps persist. Effective patient stratification, integrating disease-related risk factors into cardiovascular risk assessment tools, remains a priority. Improved awareness of the psoriasis-CVD link could enable earlier diagnosis and treatment of comorbidities, ultimately improving morbidity and mortality outcomes. Further research is essential to refine therapeutic strategies and optimize patient management (Table 4).

## 6. Ankylosing Spondylitis

Ankylosing spondylitis (AS) is a systemic rheumatic disease primarily affecting the axial skeleton, often accompanied by extra-articular manifestations such as anterior uveitis, psoriasis, and IBD [183]. Patients with AS are at a significantly increased risk of CVD, with higher rates of CVD incidence, hospitalization, and mortality compared to the general population [184,185,186,187,188]. Numerous studies have highlighted the elevated prevalence of atherosclerotic risk factors and metabolic syndrome among AS patients [124,189,190,191,192]. While traditional cardiovascular risk factors play a role, disease-specific factors, including chronic inflammation and sustained disease activity, are critical contributors to the heightened CVD risk in AS [192,193,194].

AS is associated with an atherogenic lipid profile, characterized by reduced levels of HDL-C, elevated triglycerides, a higher LDL/HDL ratio, and an increased presence of small-dense LDL (sdLDL) particles [195,196,197,198]. This dyslipidemic profile is influenced directly by disease activity, as supported by Mendelian randomization studies [199]. The EULAR acknowledges AS as a condition with an increased cardiovascular risk. However, disease-specific CV risk prediction tools are not yet available, and current recommendations suggest using national guidelines or the SCORE model for CV risk assessment [154].

Although there are no established treatment thresholds for LDL-C or specific guidelines for dyslipidemia management in AS, emerging evidence supports the cardiovascular benefits of statins beyond their lipid-lowering effects [200,201,202,203,204]. Statins have demonstrated improvements in arterial wall inflammation, endothelial function, and atherosclerosis. Some studies also suggest their potential as adjunctive therapy for controlling AS-related disease activity [204,205,206].

Non-steroidal anti-inflammatory drugs (NSAIDs) remain the first-line treatment for pain and stiffness in AS. However, their use requires caution in patients with pre-existing CV risk factors or documented CVD [154,207]. Anti-TNF-α therapies have shown promise in reducing CV morbidity and mortality in AS patients, with evidence suggesting their role in mitigating subclinical atherosclerosis through anti-inflammatory mechanisms [208,209,210,211].

In summary, heightened awareness and proactive management of cardiovascular risk in AS patients are critical. Addressing both traditional and disease-related factors may improve cardiovascular outcomes and potentially contribute to better control of AS disease activity. Further research is warranted to optimize risk stratification and explore the broader therapeutic benefits of statins and anti-inflammatory treatments in this population (Table 5).

## 7. Systemic Vasculitis

Systemic vasculitis encompasses a spectrum of inflammatory and immune-mediated diseases that affect blood vessels of varying types, sizes, and locations, leading to diverse clinical manifestations [212,213]. CV complications associated with vasculitis include myocardial ischemia, heart failure, valvular disorders, myocarditis, pericarditis, pulmonary hypertension, stroke, and thromboembolic events [213,214,215]. Patients with vasculitis, particularly those with ANCA-associated vasculitis (AAV), face markedly elevated CV morbidity and mortality, with an overall CV risk approximately 65% higher than that of the general population [213,216,217,218,219,220,221,222].

The increased CV disease burden in vasculitis arises from both traditional and disease-specific risk factors, though their relative contributions remain poorly defined [71,223]. Mechanisms underlying CV events in vasculitis include active vasculitis, vascular damage with endothelial dysfunction and procoagulant states, and accelerated atherosclerosis [224,225]. Microvascular damage and impaired microvascular function further amplify CV risk, emphasizing the critical role of microcirculation in these patients [226].

Current guidelines recommend traditional CV risk assessment tools such as the FRS, QRISK3, or SCORE. However, FRS has been shown to underestimate CV risk in AAV patients, prompting suggestions for the application of a multiplication factor to account for this limitation [25,216,227]. Additionally, the Birmingham Vasculitis Activity Score has been correlated with CV events in AAV patients, underscoring the relevance of disease-specific factors in risk stratification [228,229,230,231]. The European Vasculitis Society also advocates for the use of disease-specific risk models to enhance CV risk prediction and management [71,232].

Statins, with their anti-inflammatory and endothelial-repairing properties, hold promise for reducing CV risk in vasculitis patients [233,234]. Promising results have been observed in certain vasculitis subtypes, although evidence specific to AAV remains inconclusive, pending the outcomes of ongoing trials [235,236,237,238,239].

Achieving disease remission has been associated with reduced CV risk, highlighting the importance of effective treatment strategies for vasculitis [225]. Glucocorticoids remain the cornerstone of AAV treatment, but efforts are ongoing to mitigate their cardiometabolic side effects through dose-reduction strategies [240]. Other immunosuppressive agents show potential in managing vasculitis, but their impact on CV outcomes requires further study [241,242,243].

Despite growing recognition of the interplay between vasculitis and CV disease, additional research is essential to develop disease-specific tools for CV risk assessment, refine interventions targeting traditional risk factors, and evaluate the long-term effects of current and novel therapies on CV risk and outcomes in this patient population (Table 6).

## 8. Inflammatory Bowel Disease

IBD, encompassing Crohn’s disease (CD) and ulcerative colitis (UC), is a chronic systemic immune-mediated condition characterized by relapsing and remitting intestinal inflammation, often accompanied by a range of extraintestinal manifestations [244,245,246]. Although traditional CV risk factors are not overrepresented in IBD patients compared to the general population, these individuals face a significantly elevated risk of CV events [247,248,249,250]. This increased risk is attributed to a combination of chronic systemic inflammation, endothelial dysfunction, immune dysregulation, and gut microbiota dysbiosis [247,248,251].

Chronic inflammation in IBD plays a pivotal role in the development and progression of CVD [252]. Elevated levels of pro-inflammatory biomarkers, including interleukins, TNF-α, CRP, and serum amyloid A, have been consistently observed in IBD patients and are closely associated with increased atherosclerosis and heightened CV risk [251,252,253]. Furthermore, microvascular and macrovascular endothelial dysfunction in IBD contributes to the pathogenesis of atherosclerotic cardiovascular diseases through mechanisms such as platelet aggregation, arterial stiffening, and coagulation imbalance [251,252,254,255]. Microbiota dysbiosis, another hallmark of IBD, exacerbates CV risk by altering the composition of gut microbiota and promoting the production of proatherogenic metabolites, such as trimethylamine-N-oxide (TMAO), which play a direct role in the progression of atherosclerosis [251,256,257,258].

IBD is now recognized as a non-traditional risk factor for CV disease, necessitating accurate risk estimation that accounts for disease-specific factors, including the frequency and severity of disease flares, the extent of intestinal inflammation, and the location of disease involvement [259,260]. However, current CV risk assessment guidelines lack specific recommendations for integrating these IBD-related modifiers, which may lead to an underestimation of CV risk in this population [22,260]. While the ESC acknowledges the importance of CV risk assessment in IBD patients, specific recommendations for risk prediction remain lacking. This gap highlights the need to consider IBD patients as a high-risk group for CV complications [7,260].

Among cardiovascular medications, statins have shown potential benefits in IBD patients, including reductions in systemic inflammation, lower oral steroid requirements, and decreased colorectal cancer risk [261,262,263,264,265]. However, conflicting evidence exists regarding the effects of statins on IBD activity and flares, with some studies suggesting protective effects while others indicating no benefit or even potential adverse effects [266,267,268]. Despite their safety and reported benefits, as highlighted by the ESC, general recommendations supporting the routine use of statins for CV prevention or IBD treatment are currently unavailable due to insufficient evidence [252,269,270,271].

The impact of IBD treatments on CV risk remains complex and varies among therapeutic classes. Anti-inflammatory drugs, such as 5-aminosalicylates (5-ASA) and corticosteroids, have shown inconclusive effects on CV risk, with potential adverse outcomes including increased aortic stiffness and cardiometabolic alterations, particularly with long-term corticosteroid use [271,272,273,274,275,276,277,278,279]. Anti-TNF-α agents have demonstrated promising effects in reducing CV risk, thromboembolic events, and overall mortality in IBD patients, although their influence on lipid profiles remains uncertain [269,275,277,280,281]. Similarly, JAK inhibitors have been associated with modest and reversible lipid alterations but appear safe with respect to CV and thromboembolic risk [282,283,284,285,286].

Emerging therapies such as interleukin inhibitors (e.g., ustekinumab) and the α4β7 integrin monoclonal antibody vedolizumab have not shown a significant increase in CV events among IBD patients, although long-term data on their cardiovascular safety remain limited [287,288,289,290,291,292,293].

Overall, while the evidence confirms an increased CV risk in IBD patients, comprehensive recommendations for risk assessment and management remain sparse. A better understanding of the interplay between IBD and CV disease is crucial to developing effective primary prevention strategies. Promising avenues for investigation include therapeutic approaches targeting gut microbiota and the further evaluation of IBD-specific drugs for their potential to address both intestinal inflammation and associated cardiovascular complications (Table 7).

## 9. Human Immunodeficiency Virus (HIV) and Acquired Immunodeficiency Syndrome (AIDS)

Acquired Immunodeficiency Syndrome (AIDS) represents the advanced stage of human immunodeficiency virus (HIV) infection, characterized by progressive immunodeficiency and increased vulnerability to opportunistic infections and malignancies [294]. Early initiation of antiretroviral therapy (ART) following HIV diagnosis is critical for reducing morbidity, mortality, and transmission [295]. While ART has significantly lowered mortality and new infection rates, it has also increased the prevalence of HIV due to prolonged survival [296,297], making clinicians increasingly aware of HIV-associated cardiovascular (CV) complications and ART-related CV risk [296,298,299,300]. People living with HIV (PLWHIV) have an elevated risk of CV conditions, including heart failure, atrial fibrillation, myocardial infarction, and PAD [301,302,303,304,305,306].

Despite the high prevalence of traditional CV risk factors such as smoking, hypertension, diabetes, and dyslipidemia in PLWHIV, CV risk remains elevated even after adjusting for these factors [296,304,305,307,308]. HIV-specific factors, including latent infection, immunodeficiency, cytomegalovirus co-infection, gut microbial translocation, and chronic inflammation, exacerbate this risk [296,309,310,311]. Emerging evidence links ART to CV risk, emphasizing the need for strategies to predict and mitigate this risk [312]. Traditional CV risk assessment tools often underestimate risk in HIV patients, leading to calls for models that incorporate both traditional and HIV-related factors for more accurate predictions [27,313,314].

ART regimens, particularly those containing protease inhibitors and certain nucleoside reverse transcriptase inhibitors (NRTIs), are associated with increased CV risk, necessitating careful regimen selection [315,316,317]. Additionally, ART-associated metabolic complications—such as altered lipid profiles, insulin resistance, changes in body composition, and metabolic syndrome—contribute to heightened CV risk [296,308]. Although research on ART-related CV risk is ongoing, evidence remains inconclusive [296].

Effective management strategies for PLWHIV include smoking cessation, lifestyle modifications, and aggressive management of traditional CV risk factors [308,318,319]. The 2019 ACC/AHA guidelines recognize HIV infection as a CVD risk-enhancing factor [320], but consensus on lipid-lowering therapy for PLWHIV not meeting statin therapy indications remains elusive [321]. The ESC guidelines for dyslipidemia management recommend LDL-C targets defined for high-risk patients, with statins as the preferred first-line therapy [22]. Pravastatin, fluvastatin, pitavastatin, and rosuvastatin have demonstrated favorable effects in PLWHIV, provided potential drug interactions are managed [22]. Statins exhibit anti-inflammatory and immunomodulatory effects in PLWHIV, including reductions in vascular inflammation markers and diminished T-cell and monocyte activation [318,319,320,321]. However, further research is needed to determine their impact on CV risk modulation [322,323,324,325,326]. For patients intolerant to statins or unable to achieve LDL-C targets, Ezetimibe may be a viable option despite its limited efficacy [327]. PCSK9 inhibitors offer a safe and effective alternative for high-risk patients unable to reach therapeutic goals [328], while fibrates and fish oils are reserved for managing hypertriglyceridemia [329,330].

In conclusion, PLWHIV face a significantly higher CV risk that requires comprehensive stratification and management. Understanding the interplay between traditional and HIV-associated CV risk factors is critical for accurate risk prediction and targeted prevention strategies. Proactive management of modifiable risk factors, early initiation of lipid-lowering therapy, and careful selection of ART regimens with favorable CV profiles are essential to mitigating cardiovascular disease in PLWHIV (Table 8).

## 10. Cancer

Cancer represents a diverse group of malignancies characterized by the uncontrolled proliferation of genetically altered cells, leading to local invasion and metastasis [331].

Alongside CVD, cancer imposes a significant global burden, contributing to high rates of morbidity and mortality [332]. Cancer patients not only face the challenges of their malignancy but also exhibit a heightened risk of developing CVD and experiencing CVD-related mortality compared to the general population [333,334,335,336,337]. Advances in cancer therapies have improved survival rates but have also increased the competing risk of CVD-related deaths [21,335].

Emerging evidence highlights a bidirectional interplay between cancer and CVD, positioning cancer as a non-traditional risk factor for CVD [21]. Shared pathogenic mechanisms include traditional CVD risk factors (e.g., diet, physical inactivity, hypertension, diabetes, obesity, smoking, and social determinants of health) and dysregulated processes such as inflammation, immune response, oxidative stress, metabolism, hormonal changes, gut microbiome alterations, and genetic factors [21,338,339,340,341,342,343,344,345,346,347].

Effective CV risk stratification in cancer patients is crucial for managing risk factors, implementing preventive strategies, and tailoring follow-up programs. However, current CV risk assessment tools often fail to account for cancer and its treatments, resulting in underestimation of overall CV risk [28,29,348,349]. This gap highlights the need for cancer-specific CV risk stratification models and corresponding therapeutic guidelines, particularly for tailored lipid-lowering strategies [7,22].

Statins, traditionally used for their CV benefits, have shown promise in cancer prevention and adjunctive treatment. Dyslipidemia is linked to carcinogenic processes, including tissue invasion and metastasis, making cholesterol biosynthesis inhibition a potential anti-cancer mechanism of statins [350,351,352,353,354]. Beyond cholesterol-related pathways, statins exhibit pleiotropic effects [350,352,355,356,357,358], including modulation of proliferation, apoptosis, autophagy, angiogenesis, tumor metastasis, the tumor microenvironment, and drug resistance [350,359,360,361,362,363,364,365,366,367]. Studies have reported positive associations between statin use and cancer outcomes, such as reduced cancer risk [368,369,370,371], lower cancer grade and stage at diagnosis [372,373], and improved survival rates, including reduced overall and progression-free mortality [374,375,376,377,378].

Despite these promising findings, the evidence on statins as anti-cancer agents remains inconsistent, and well-designed clinical trials are needed to clarify their role [356,379,380,381].

The well-documented relationship between cancer and CVD underscores the urgent need for comprehensive and targeted CV risk assessment in oncology patients. While the repurposing of statins as part of cancer treatment is gaining attention, the magnitude of their benefit remains debated. Further research is essential to refine CV risk management and explore the therapeutic potential of statins in cancer care (Table 9).

## 11. Discussion

Despite substantial advancements in preventing and treating traditional CV risk factors through the use of highly effective clinical tools and evidence-based therapeutic strategies, CV events continue to represent a major challenge in patients with atherosclerotic diseases [382,383]. This phenomenon, referred to as residual CV risk, highlights the persistence of CV events even when treatment goals for traditional risk factors are achieved. A significant component of this residual risk is residual inflammatory risk (RIR), characterized by an ongoing pro-inflammatory response that contributes to the progression of atherosclerosis and CV events [384,385,386]. As a result, targeting inflammatory pathways has emerged as a promising approach to achieving more comprehensive CV risk control [387,388,389].

Patients with “non-traditional” atherosclerotic conditions, such as RA, SLE, SSc, ankylosing AS, systemic vasculitis, psoriasis, and HIV infection, are at an increased risk of CV events, morbidity, and mortality [21,22]. While these patients exhibit a higher prevalence of traditional atherosclerotic risk factors, including hypertension, dyslipidemia, diabetes, and smoking, these factors alone do not fully explain the heightened CV risk observed in these populations [21,34,36,60,71,99,106,118,152,192,225,299,308,390]. Chronic inflammation plays a central role in driving and sustaining atherosclerosis in these conditions, often interacting synergistically with traditional risk factors to exacerbate the inflammatory burden. This interplay creates a vicious cycle that significantly amplifies CV risk [31,62,192,252,338,339,391].

In addition to chronic inflammation, a variety of disease-specific factors contribute to the elevated CV risk seen in “non-traditional” atherosclerotic diseases. For example, microvascular dysfunction and impaired microvascular dynamics in systemic sclerosis and systemic vasculitis are key contributors. IBD and cancer are associated with microbiota dysbiosis, which promotes CV risk by altering the gut microbiome and generating pro-atherogenic metabolites. Cancer is further complicated by oxidative stress, metabolic and hormonal dysregulation, and immune system alterations. Similarly, in HIV infection, ART contributes to CV risk through metabolic changes and persistent inflammation, despite its life-saving benefits [99,106,224,251,299,341,344,345,347]. Moreover, for almost the whole of the aforementioned conditions, a disease activity- and severity-dependent relationship with CV risk has been recognized [22,73,138,142,228,229,230].

Given the multitude and the complexity of involved factors, a multidisciplinary approach including immunologists, gastroenterologists, rheumatologists, and infectious disease specialists is the cornerstone for the management of this heterogeneous population. All clinicians should support the cardiologist in developing tailored interventions to enhance patient outcomes.

Despite the increased CVD risk in “non-traditional” atherosclerotic diseases, more and more attention has been paid by the scientific community to the absence of specific guidelines for CV risk management in these conditions, which often leads to suboptimal care [7,22,23,28,29,71,121,153,154,216]. Specifically, two core improvement areas can be identified: CV risk estimation and CV risk factor treatment.

Current risk assessment tools, which rely on traditional risk factors, frequently underestimate CV risk in these populations, underscoring the need for disease-specific models that account for disease-related variables [22,28,29,73,260,313]. In RA, LES, and systemic vasculitis and psoriasis patients, the attempt to apply a multiplication factor to compensate for the traditional tools is documented to result in a CV risk underestimation [23,121,153,216]. Therefore, disease-specific tools, including disease-related variables, particularly those regarding disease activity and severity, have been proposed [22,73,260,313]. However, due to limited evidence and their pending validation, guidelines recommend performing CV risk stratification making use of general population-aimed tools [7,71,121,154]. The only exceptions are the ESC position paper on lipid management in rheumatoid arthritis and the European Vasculitis Society model for systemic vasculitis [22,71].

Advanced imaging and novel biomarkers may be key to overturning CV risk stratification and treatment, ultimately reducing CV morbidity and mortality. Systemic inflammation markers, including hsCRP (high-sensitivity C-reactive protein), IL-6 (interleukin-6), and TNF-α (tumor necrosis factor-α), provide incremental prognostic information on top of clinical risk models [392,393,394,395,396,397,398]. However, due to the lack of specificity and direct causal association with CV disease, they appear to have limited predictive power. The advent of more advanced imaging technologies, including ^18^F-FDG (18F-fluorodeoxyglucose) for glucose uptake, ^68^Ga-DOTATATE (gallium-68 DOTATATE) for M1 macrophages, ^18^FNaF (18F-sodium fluoride), PCATa (pericoronary adipose tissue attenuation), and CCTA-HRP (coronary computed tomography angiography–high-risk plaque), offers alternative ways to quantify vascular inflammation and maximize the prognostic value of biomarkers [399,400,401,402,403,404,405,406,407,408,409].

Optimal CV risk factor treatment stems from accurate risk factor stratification: estimating CVD risk provides mandatory information for tailored intervention. The lack of clear indications regarding risk stratification and traditional risk factor prevention and treatment results in a new paradox that could be defined as “Residual Traditional Risk” (RTR).

In terms of prevention and lipid management, with the sole exception of HR-RA patients, no disease-specific dyslipidemia recommendations or LDL-c disease-specific targets have been identified. The overall benefits recorded with statin therapy appear to be greater than what could be expected from the sole change in lipid profile, therefore advocating for “cholesterol-independent” effects. As a result, in line with a growing body of evidence suggesting a potential effect on disease activity, a new paradigm for statin therapy has been advocated, and statin therapy is now experiencing a new burst in “non-traditional” atherosclerotic conditions [26,38,41,42,43,74,76,84,85,114,115,116,203,205,206,239,261,322,323].

As discussed above, part of the overall residual CV risk can be attributed to residual inflammatory risk [384,385,386]. This raises the question of whether the anti-inflammatory action of disease-specific medications results in a reduced CV risk. Although disease-specific treatments may have anti-inflammatory effects with the potential to lower CV risk, evidence supporting their efficacy for this purpose remains limited [46,89,90,167,169,174,179,208,210,240,241,242,243,269,272,275,282,285,287,288,289,410]. However, controlling inflammation has been shown to standardize CV risk and improve prevention and treatment outcomes [46,89,90,167,169,174,179,208,210,240,241,242,243,269,272,275,282,285,287,288,289,392]. In light of the above, disease therapy should be considered as a CV risk modulator advocating a new paradigm for disease-specific medications too.

For patients with HIV, ART is essential for managing the infection but increases CV risk due to its effects on metabolism and inflammation [308,411,412]. Despite this, the overall benefits of ART outweigh the associated CV risks, although monitoring CV risk and prioritizing “lipid-friendly” ART regimens are essential strategies [319].

**Figure 1:**
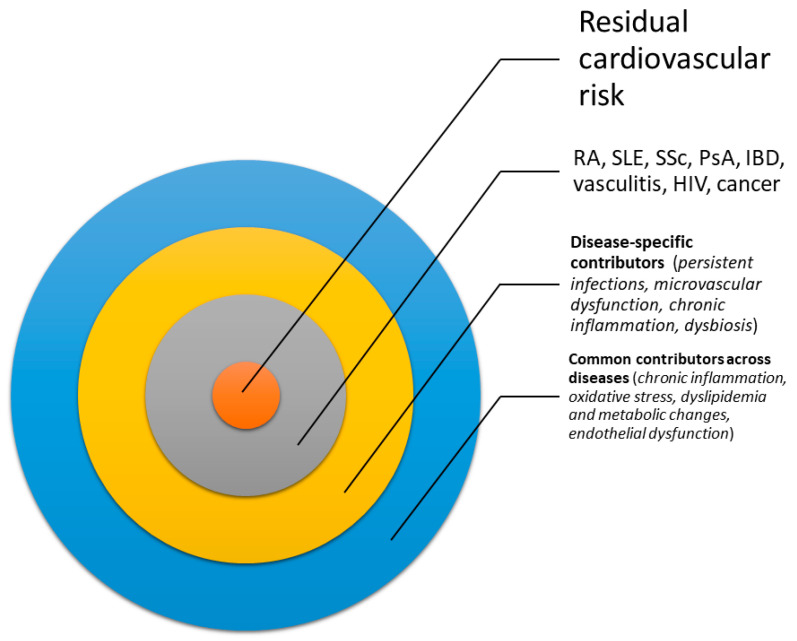
The figure illustrates the multifactorial residual cardiovascular (CV) risk in non-traditional atherosclerotic conditions. The central circle represents persistent residual CV risk despite traditional risk management. The inner layer lists associated diseases [e.g., rheumatoid arthritis (RA), systemic lupus erythematosus (SLE), systemic sclerosis (SSc), psoriasis and psoriatic arthritis (PsA), HIV, vasculitis, and inflammatory bowel disease (IBD)]. The middle layer highlights disease-specific contributors, including infections, microvascular dysfunction, and metabolic dysregulation. The outer layer shows shared risk factors like chronic inflammation, oxidative stress, dyslipidemia, and endothelial dysfunction.

The interplay between these factors in amplifying CV risk is represented in Figure 1.

## 12. Conclusions

In recent decades, the scope of conditions requiring meticulous management of CV risk factors has broadened, now encompassing several “non-traditional CV risk-enhancer conditions”. While traditional risk factors have historically been the cornerstone of CV prevention strategies for “traditional atherosclerotic conditions”, the growing recognition of inflammation’s critical role in atherosclerosis has shifted focus toward residual inflammatory risk as a major contributor to overall CV risk. In contrast, for “non-traditional atherosclerotic conditions”, inflammation has always been central to disease-specific treatments, though the elevated CV risk associated with these conditions has only recently been fully appreciated.

Despite increased clinical research in this area, significant gaps remain regarding the interplay between traditional and disease-specific risk factors, the accurate assessment of overall CV risk, and the optimal management of traditional risk factors in these conditions. In “non-traditional CV risk-enhancer conditions”, traditional risk factors function as residual risk contributors, much like the residual “traditional” risk observed in patients with traditional atherosclerotic conditions.

Unlike patients with “traditional atherosclerotic conditions”, those with “non-traditional” conditions often have their first medical encounter well before a CV event occurs. This early engagement provides a unique opportunity to implement tailored interventions during the subclinical stages of CV disease. As such, clinician awareness of “non-traditional CV risk-enhancer conditions” is essential for achieving a holistic understanding of these conditions and ensuring effective CV risk management.

With the provoking introduction of the concept of “Residual traditional risk”, this review aims to stimulate debate within the scientific community and foster dialogue among clinicians in order to identify solutions to address the emerging needs and update clinical guidelines and public health policies.

Future research should focus on developing comprehensive CV risk prediction models that integrate disease-specific risk factors alongside traditional ones. The identification of disease-specific risk factors, the assessment of each risk factor’s magnitude, and how disease-specific risk factors influence the underlying CV risk represent key elements of their development roadmap.

Accurate CV risk estimation in these populations could lead to more targeted and effective management strategies, ultimately reducing CV morbidity and mortality. By bridging existing knowledge gaps and enhancing risk prediction, these efforts have the potential to transform the care of patients with “non-traditional CV risk-enhancer conditions”.

## Figures and Tables

**Table 1:** Key findings in rheumatoid arthritis.

	Summary of Evidence	Articles
CV risk and CV outcomes	Chronic inflammatory burden is central to atherosclerosis, leading to heightened CV risk, morbidity, and mortality in RA patients.	[31,32,33,34,35,36]
CV risk stratification	Existing tools and traditional calculators often misestimate CV risk in RA patients. Including disease-specific factors is essential for accurate stratification.	[22,23]
Lipid lowering therapy	Statins not only lower LDL levels but also exhibit pleiotropic effects, making them valuable for CV risk reduction and adjunctive RA disease control.	[37,38,39,40,41,42,43]
Disease therapy	DMARDs provide cardiovascular protection. Biologic agents, including anti-TNF, IL-6 inhibitors, and JAK inhibitors, reduce CV morbidity and events.	[34,46,47,52,53,54,55]

**Table 2:** Key insights into systemic lupus erythematosus.

	Summary of Evidence	Articles
CV risk and CV outcomes	Both traditional and disease-specific factors contribute to the elevated risk of CV events and mortality in SLE. SLE is recognized as an independent risk factor for CVD and classified as a “CVD risk equivalent”.	[60,61,70]
CV risk stratification	Enhancing risk estimation models requires incorporating additional biomarkers and disease-specific risk factors, as current tools fall short in addressing the complexity of SLE-related CV risk.	[23,73]
Lipid lowering therapy	Statins demonstrate significant benefits in reducing CV risk, morbidity, and mortality. Emerging evidence suggests they may also influence disease activity.	[74,75,76,77,78,84,85,95]
Disease therapy	Targeting inflammatory pathways to suppress disease activity offers a promising approach for reducing cardiovascular risk in SLE patients.	[86,87,88,89,90,91,92,93,94]

**Table 3:** Summary of evidence in systemic sclerosis.

	Summary of Evidence	Articles
CV risk and CV outcomes	SSc patients exhibit a higher prevalence of macrovascular diseases, suggesting a potential increased risk of CVD.	[98,99,100,101]
CV risk stratification	Comprehensive CV risk evaluation, including microvascular impairment, remains underutilized. Current risk stratification relies on tools designed for the general population.	[24,71]
Lipid lowering therapy	While evidence highlights the benefits of statins in preventing endothelial dysfunction, no specific recommendations have been established for their use in SSc patients.	[114,115,116]

**Table 4:** Key Findings in psoriasis and psoriatic arthritis.

	Summary of Evidence	Articles
CV risk and CV outcomes	Psoriasis is now recognized as an independent risk factor for CVD, with the higher prevalence of CVD not fully explained by traditional cardiovascular risk factors.	[111,119,120,121,122,123,124,125,126,127,128,129,130,133,134,135]
CV risk stratification	Current clinical tools perform suboptimally as they exclude non-traditional risk factors. The Joint AAD advocates for adjustments to risk scoring tools, while similar recommendations are absent in AHA guidelines.	[26,121,151]
Lipid lowering therapy	Although studies show promising results, the inconsistent findings on lipid-lowering therapy efficacy in psoriasis treatment highlight the need for more robust evidence.	[26,155,156,157,158,159,160,161,162,163,164,165,166]
Disease therapy	Anti-psoriatic drugs show potential benefits in reducing CVD risk and metabolic comorbidities, with ongoing research yielding encouraging results.	[167,168,169,170,171,172,173,174,175,176,177,178,179,182]

**Table 5:** Summary of evidence in ankylosing spondylitis.

	Summary of Evidence	Articles
CV risk and CV outcomes	While AS patients have a higher prevalence of atherosclerotic risk factors, disease-related mechanisms such as chronic inflammation and disease activity significantly contribute to the elevated CVD risk.	[124,189,190,191,192,193,194]
CV risk stratification	EULAR acknowledges AS as a condition with increased CV risk but does not provide disease-specific CVD risk prediction tools, recommending the use of national guidelines or the SCORE model.	[154]
Lipid lowering therapy	Statins, due to their pleiotropic effects, are gaining attention in AS management, not only for cardiovascular protection but also as potential adjunctive therapy to help control disease activity.	[200,201,202,203,204,205,206]
Disease therapy	While NSAIDs remain the first-line treatment for AS symptoms, their use requires caution in patients with CV risk factors or CVD history. Anti-TNF-α therapies have demonstrated reductions in CV morbidity and mortality.	[154,207,208,209,210,211]

**Table 6:** Key findings in systemic vasculitis.

	Summary of Evidence	Articles
CV risk and CV outcomes	CV disease in vasculitis patients is driven by both traditional and disease-specific factors, including inflammation and vascular damage.	[71,213,216,217,218,219,220,221,222,223]
CV risk stratification	Traditional tools underestimate CV risk. Guidelines recommend integrating disease-specific models for improved assessment.	[25,71,216,227,232]
Lipid lowering therapy	Statins show potential due to anti-inflammatory and endothelial-repairing effects, but specific recommendations are lacking.	[233,234,235,236,237,238,239]
Disease therapy	Disease remission reduces CV risk, but evidence on immunosuppressive agents and CV outcomes remains inconclusive.	[225,241,242,243]

**Table 7:** Key findings in inflammatory bowel disease.

	Summary of Evidence	Articles
CV risk and CV outcomes	Increased CV risk in IBD is driven by traditional risk factors, chronic inflammation, endothelial dysfunction, and microbiota dysbiosis.	[247,248,249,250,251]
CV risk stratification	Guidelines lack clear recommendations on integrating IBD-specific factors, leading to potential CV risk underestimation.	[22,259,260]
Lipid lowering therapy	Statins show benefits, including reduced inflammation and steroid use, but lack general recommendations for IBD prevention or treatment.	[252,261,262,263,264,265,269,270,271]
Disease therapy	Anti-inflammatory drugs may reduce ASCVD risk, but evidence across drug classes remains inconsistent.	[269,271,272,273,274,275,276,277,278,279,281,282,283,284,285,286,287,288,289,290,291,292,293]

**Table 8:** Summary of evidence in acquired immunodeficiency syndrome (AIDS).

	Summary of Evidence	Articles
CV risk and CV outcomes	Increased CVD risk in HIV patients remains significant even after adjusting for traditional CV risk factors, with HIV-specific factors further contributing to the risk.	[296,304,305,307,308,309,310]
CV risk stratification	General population risk tools underestimate CVD risk in PLWHIV. Models incorporating both traditional and HIV-specific factors may improve risk prediction accuracy.	[313]
Lipid lowering therapy	There is no consensus on the benefits of lipid-lowering therapy for PLWHIV not meeting current statin therapy indications.	[321]
Disease therapy	ART regimens, particularly those with protease inhibitors or certain NRTIs, increase CVD risk. Careful selection of “lipid-friendly” ART regimens is strongly recommended.	[308,315,316,317,319]

**Table 9:** Key findings in cancer.

	Summary of Evidence	Articles
CV risk and CV outcomes	Cancer patients face an increased risk of CVD and CVD-related mortality, influenced by both the disease and its treatments.	[333,334,335,336,337]
CV risk stratification	Current CV risk tools do not account for cancer or cancer treatments, leading to underestimation of overall CV risk.	[28,29,348,349]
Lipid lowering therapy	While promising results have been observed, clear recommendations for lipid-lowering therapy as an adjunctive treatment in cancer patients are lacking.	
